# Sodium houttuyfonate effectively treats acute pulmonary infection of *Pseudomonas aeruginosa* by affecting immunity and intestinal flora in mice

**DOI:** 10.3389/fcimb.2022.1022511

**Published:** 2022-12-01

**Authors:** Tian Zhuang, Mengxue Hu, Jian Wang, Longfei Mei, Xiaoxiao Zhu, Haitao Zhang, Feng Jin, Jing Shao, Tianming Wang, Changzhong Wang, Xiaojia Niu, Daqiang Wu

**Affiliations:** ^1^ Department of Pathogenic Biology and Immunology, College of Integrated Chinese and Western Medicine, Anhui University of Chinese Medicine, Hefei, China; ^2^ Research Institute of Integrated Traditional Chinese and Western Medicine, Anhui Academy of Chinese Medicine, Hefei, China; ^3^ Key Laboratory of Xin’an Medicine, Ministry of Education, Anhui University of Chinese Medicine, Hefei, China; ^4^ Pathology Department, First Affiliated Hospital of Anhui University of Chinese Medicine, Hefei, China

**Keywords:** Sodium houttuyfonate, *Pseudomonas aeruginosa*, inflammatory factor, TLR4/NF-κB pathway, intestinal flora

## Abstract

**Introduction:**

*Pseudomonas aeruginosa* is a major nosocomial pathogen that frequently causes ventilator-associated pneumonia in specific populations. Sodium houttuyfonate (SH) has shown mild antibacterial activity against *P. aeruginosa in vitro*, but the mechanism of potent antimicrobial activity of SH against *P. aeruginosa* infection *in vivo* remains unclear.

**Methods:**

Here, using the mouse pneumonia model induced by *P. aeruginosa* nasal drip to explore the therapeutic effects of SH.

**Results:**

We found that SH exhibits dose-dependent therapeutic effects of reducing *P. aeruginosa* burden and systemic inflammation in pneumonia mice. SH ameliorates inflammatory gene expression and production of inflammatory proteins, such as interleukin-6 (IL-6), nuclear factor kappa-B (NF-κB) and toll-like receptor 4 (TLR4), associated with the TLR4/NF-κB pathway in mice with *P. aeruginosa* pneumonia. Furthermore, we analyzed the intestinal flora of mice and found that compared with the model group, the abundance and diversity of beneficial bacterial flora of SH treatment groups increased significantly, suggesting that SH can improve the intestinal flora disorder caused by inflammation. In addition, SH improves alpha and beta diversity index and reduces species abundance differences of intestinal flora in pneumonia mice.

**Discussion:**

Taken together, our presented results indicate that SH may effectively alleviate the acute pulmonary infection induced by *P. aeruginosa* by reducing the disturbance of regulating immunity and intestinal flora in mice.

## Introduction

*P. aeruginosa* is a common conditional pathogen in clinic, which is easy to cause respiratory tract infection and a series of severe suppurative conditions in immunodeficiency patients (Pang et al., 2019). Due to the high level of intrinsic and acquired drug resistance of *P. aeruginosa*, the infection caused by it usually cannot get targeted treatment (Morton et al., 2014). Thus, it is imperative to find new therapeutic drugs and their targets.

*Houttuynia cordata* Thunb has long been applied as an antipyretic and detoxifying traditional Chinese herbal drug and is widely used to treat infection because of its superior protective and antibacterial effects in East and Southeast Asia (Liu et al., 2021). There are several medicinal reactive chemical components of *Houttuynia cordata*, mainly composed of alkaloids, organic acids, volatile oil, polysaccharides, etc. Houttuynin is the key component in the volatile oil of the main extract of *Houttuynia cordata*. The dutasteride of houttuynin is decanoyl acetaldehyde, but is instability. Sodium houttuyfonate (SH), which is an addition compound of sodium bisulfite and houttuynin, can overcome the chemical instability of houttuynin. SH is extensively applied in antimicrobial clinic areas in China as an alternative drug, which has the same pharmacological effects but is more stable and soluble compared with houttuynin (Wang et al., 2017). Now, there are numerous scientific evidences supporting that SH appears to exhibit selectivity for lung tissues, such as alleviating pneumonia (Wu et al., 2017), lung injury (Liu et al., 2021), pulmonary fibrosis (Shen et al., 2021). Previously, we found that SH can inhibit biofilm formation and alginate production in *P. aeruginosa* (Wu et al., 2015; Wang et al., 2019), and effectively suppress the production of virulence factors regulated by motility and Las quorum sensing systems associated with its pathogenicity (Wu et al., 2014). Additionally, results from *in vivo* studies showed that the combination of SH and EDTA-Na_2_ extended the lifespan of animals infected with *P. aeruginosa* (Huang et al., 2015; Wu et al., 2015). These results imply that SH can affect the growth and physiological functions of *P. aeruginosa* through a variety of mechanisms, but the mechanisms by which it exerts its therapeutic effects *in vivo* need to be further investigated.

Inflammation and repair in the human body are usually accompanied by an increase in chemokines, cytokines, and growth factors (Shen et al., 2021). Variations in inflammatory cytokine levels have been considered to play a crucial role in the inflammatory response caused by a bacterial infection (Tran et al., 2019). Furthermore, in recent years, there is increasing evidence that the TLR4/NF-κB signaling pathway can be involved in the immune response to various diseases by regulating the expression of inflammatory factors such as interleukin-6 (IL-6), interleukin-1β (IL-1β) and tumor necrosis factor-α (TNF-α) (Chen et al., 2018; Su et al., 2018). Therefore, TLR4/NF-κB signaling pathway is likely to be an important strategy for the treatment of *P. aeruginosa* infection. In addition, the intestinal flora is also closely related to the immune response *in vivo*, especially changes in the composition and function of microorganisms in the respiratory and intestinal tracts that can influence the immune response and lung diseases (Ratner et al., 2005; Bernasconi et al., 2016). In a previous study, we found that high doses of SH and sodium new houttuyfonate (SNH) can affect the composition of the intestinal flora and the production of inflammatory factors in healthy KM mice (Yan et al., 2020). However, the mechanism of action of SH to improve lung inflammation and intestinal flora in mice’s acute pulmonary infection is unclear and worth to investigate.

Therefore, in this study, we utilized a mouse model of acute lung infection induced by drug-resistant *P. aeruginosa* by nasal drip (Allewelt et al., 2000; Koh et al., 2009; Wu et al., 2012) and investigated how SH affects lung pathology, bacteriology, serum inflammatory factors, expression of relevant immune pathways, and changes in intestinal flora. Our presented results will provide a new perspective and experimental insight to guide clinical utilization.

## Materials and methods

### Chemicals, animals and bacterial strains

Sodium houttuyfonate (SH) was purchased from Xi’an Kai Lai Biological Engineering Co., Ltd (Xi’an, China, code No. K196584), with the purity≥ 98%. Azithromycin (AZM) was acquired from the Northeast Pharmaceutical Group Shenyang No. 1 Pharmaceutical Co., Ltd. (Shenyang, China). Ninety specific pathogen-free (SPF) BALB/c mice (male, 6 – 8 weeks, 20 ± 5 g) provided by (Hefei, China, license No. SCXK Anhui 2017-001) were housed at 18 – 25°C and 50 – 70% relative humidity, under a 12h light-dark cycle. The animal ethics committee of Anhui University of Traditional Chinese Medicine approved the animal experiments and provided ethical proof documents. *P. aeruginosa* strain ATCC 27853 (Gao et al., 2011), which was purchased from the National Institute for the Control of Pharmaceutical and Biological Products (NICPBP, Beijing, China).

### Animal model establishment and drug treatment

After seven days of adaptive feeding, 90 mice were randomly and averagely divided into control, model, high dosage (SH 100 mg/Kg), moderate dosage (SH 50 mg/Kg), low dosage (SH 25 mg/Kg) and azithromycin (AZM 12 mg/Kg) groups based on our previous research, and 15 mice per group.

Based on mouse model established by Allewelt and colleagues (Allewelt et al., 2000), weighing mice were anesthetized with 1% pentobarbital sodium (dosage of 40mg/kg). While the mice were in an upright position, a micro sampler was used to inject 10μl (a total of 20μl) of fresh bacterial liquid (1.5 × 10^8^ CFU/ml) into each nostril to maintain an upright position of about 1min to ensure that the bacterial liquid entered the airway and distributed as evenly as possible in both lungs. According to the above method, the control group was given nasal drops with the same amount of normal saline once a day, and repeated for seven days. Seven days later, the mice in each group were given intragastric administration according to each dose, and the control group was assigned normal saline once a day, and repeated for seven days. The eating behavior, hair, mental state and survival state of mice in each group were observed every day, and the bodyweight changes were recorded. At fourteenth day, anesthesia was performed with 3% pentobarbital sodium and humanitarian execution according to animal ethics requirement of the Animal Ethics of Committee of our university.

### Hematoxylin-eosin staining

The right lungs of three mice from each group were isolated in a sterile environment, and the lungs were immediately flushed with 4% paraformaldehyde. After flushing, the lungs were fixed in 5 times the volume of 4% paraformaldehyde buffer for at least 72 hours. After dehydration and paraffin embedding, 5mm lung tissue sections were taken for Hematoxylin-eosin (H & E) staining. The pathological changes of lung tissue were observed under an upright microscope (OLYMPUS BX51, Tokyo, Japan). The pathological change degree scoring method is based on the literature (Liu et al., 2004).

### *P. aeruginosa* burden assessment

Left lung tissue from nine mice in each group was isolated in a sterile environment, and the plate counting method was used to detect the number of *P. aeruginosa* in the lung tissue. The lung tissue samples were weighed and 2ml phosphate buffered saline (PBS) per gram of lung tissue was added to prepare the lung tissue homogenate in an ice bath at 4 °C. The tissue homogenate was diluted to 10^3^ with a gradient of 10 times concentration (if the colony was too dense, it would continue to be diluted). 50μL of homogenate diluent of each concentration was smeared evenly on 9 cm Luria-Bertani (LB) agar plate with a glass rod and repeated 3 times at each concentration. The plates were cultured in 37°C incubator for 24 hours, and the colonies were counted to calculate CFU/g lung tissue.

### Spleen index

The spleen tissue of each mouse was isolated in an aseptic environment. The body weight and spleen weight of each group was measured, and the spleen index was calculated by the equation of “spleen index = spleen mass (mg)/mouse weight (g)”.

### ELISA analysis

The peripheral blood samples were collected by the eyeball blood collection method and centrifuged at the speed of 5000 r/min for 10 minutes, and the serum was separated. Cytokines in serum, including IL-6 (Jianglai, Shanghai, China, #JL20268), C-X-C motif chemokine ligand 1 (CXCL1) (Jianglai, Shanghai, China, #JL20271), and interleukin-17 (IL-17) (Jianglai, Shanghai, China, #JL20250), IFN-γ (Jianglai, Shanghai, China, #JL10967), IL-25 (Jianglai, Shanghai, China, #JL31907) were measured separately with ELISA kits according to the instructions provided by the manufacturer.

### qRT-PCR

The right lungs of six mice from each group were isolated in a sterile environment. Total RNA was extracted from lung tissue using Trizol reagent (Ambion, USA). FastQuant RT Kit reverse transcription kit (TIANGEN, Beijing, China) was used to reverse RNA into complementary DNA (cDNA), and the genomic DNA were removed by the DNAase in the kit. *IL-1β*, *IL-6*, *interleukin-10* (*IL-10*), *Nuclear Factor kappa B* (*NF-κB*), *TNF-α*, *Interferon-γ* (*IFN-γ*) and *Toll−like receptor 4* (*TLR4*) genes were quantitatively amplified by qRT-PCR using primers listed in **Table 1**. The primers were designed by Primer Premier 5.0 and synthesized by Sangon Biotech (Shanghai, China). SYBR Green real-time PCR Master Mix (TOYOBO, Tokyo, Japan) was performed in the ABI7000 fluorescence quantitative PCR system (Thermo Fisher, USA). The fold change of expression of each gene was quantitatively calculated by the 2^-ΔΔCT^ method with *β-actin* as the reference gene.

**Table 1:** Primers for qRT-PCR.

Oligo name	Sequence (5’ to 3’)	Product size (bp)
*β-actin* Forward	CGT AAA GAC CTC TAT GCC AAC A	163
*β-actin* Reverse	AGC CAC CAA TCC ACA CAG AG	
*IL-1β* Forward	CAA CCA ACA AGT GAT ATT CTC CAT G	152
*IL-1β* Reverse	CAT TCT GTC TCG AGC CCA CC	
*IL-6* Forward	GCT GGA AGT CTC TTG CGG AG	80
*IL-6* Reverse	GCT GGA AGT CTC TTG CGG CG	
*IL-10* Forward	ACT GGC ATG AGG ATC AGC AG	350
*IL-10* Reverse	AGA AAT CGA TGA CAG CGC CT	
*NF-κB* Forward	CCT CTC TCG TCT TCC TCC AC	94
*NF-κB* Reverse	GTT TGC GGA AGG ATG TCT CC	
*TNF-α* Forward	GCA TGA TCC GAG ATG TGG AAC TGG	113
*TNF-α* Reverse	CGC CAC GAG CAG GAA TGA GAA G	
*IFN-γ* Forward	GAG TAT TGC CAA GTT TGA GGT	123
*IFN-γ* Reverse	CAG CGA CTC CTT TTC CGC T	
*TLR4* Forward	GTT CTC TCA TGG CCT CCA CT	123
*TLR4* Reverse	GCA GGG ATT CAA GCT TCC TG	

### Western blotting

The right lungs of six mice from each group were isolated in sterile environment and used to extract total protein. The total protein was extracted with radioimmunoprecipitation assay (RIPA) (SparkJade, Shandong, China) cleavage buffer containing phenylmethanesulfonyl fluoride (PMSF) (SparkJade, Shandong, China) and phosphatase inhibitor cocktail A (Beyotime, Shanghai, China). Bicinchoninic acid (BCA) protein analysis kit (SparkJade, Shandong, China) was used to determine the protein concentration of each group. The 20μg total protein was subjected to SDS-PAGE gel electrophoresis, then transferred to the polyvinylidene difluoride (PVDF) membrane (Immobilon-P, USA), and sealed in 5% skimmed dried milk, then incubated overnight with the first antibody at 4°C: NF-κB p65 Rabbit mAb (1:1000; ZENBIO, Chengdu, China, #310126); Phospho-NF-κB p65 Ab (1:1000; Affinity, Jiangsu, China, #AF2006); IκB alpha Ab (1:1000; Affinity, Jiangsu, China, #AF5002); Phospho-IκB alpha Ab (1:1000; Affinity, Jiangsu, China, #AF2002); Toll-Like Receptor 4 Rabbit pAb (1:1000; ZENBIO, Chengdu, China, #505258); IL-1 beta Rabbit pAb (1:1000; ZENBIO, Chengdu, China, #511369); TNF alpha Rabbit pAb (1:1000; ZENBIO, Chengdu, China, #346654); Interferon gamma Rabbit pAb (1:1000; ZENBIO, Chengdu, China, #381656); beta Actin Mouse mAb (1:10000; ZENBIO, Chengdu, China, #200068-8F10). The membrane was washed with Tween-20/Tris buffered saline (TBS) 3 times, each 10 minutes, and then incubated with the second antibody (1:2000; Elabscience, Wuhan, China, #E-AB-1003) at room temperature for 1 hours. After washing with Tween 20/TBS 3 times, the developer treated the membrane (SparkJade, Shandong, China) and observed and photographed it by an ECL imaging system (LAS4000, GE, Pittsburgh, PA, USA). Image J (National Institutes of Health, Bethesda, Maryland, USA) was used to measure the density of the data, and the density measurement data were normalized by internal reference protein β-actin.

### Illumina MiSeq sequencing

To evaluate the structure of intestinal flora in mice, fecal samples were collected in a sterile environment after the completion of treatment. To reduce the difference within the group, 12 fecal samples from each group were randomly selected and merged into 6 samples in sterile microcentrifuge tubes, which were quickly placed in dry ice and immediately sent to Majorbio Biopharmaceutical Technology Co., Ltd. (Shanghai, China) for microbial DNA extraction.

Equimolar and peer-to-peer sequencing (2 × 300 bp) was performed on the Illumina MiSeq PE300 platform (Illumina, San Diego, USA) according to the standard protocol of Majorbio Bio-Pharm Technology (Shanghai, China). The original sequencing data were stored in the NCBI Sequence Read Archive (SRA) database (Accession Number: PRJNA781056). The original 16s rRNA gene sequencing reads was filtered by fastp and merged by FLASH. The criteria are as follows (Di Segni et al., 2018): (1) in a sliding window of 50 bp, In a sliding window of 50 bp, reads with an average mass fraction of less than 20 were intercepted and overlapping sequences smaller than 50 bp and containing ambiguous characters were merged with the reads; (2) greater than 10bp are discarded. The maximum mismatch rate of the overlapping region is 0.2. (3) distinguish the samples according to the perfectly matched bar code and primers (allowing 2 nucleotides mismatch) and adjust the sequence direction.

The operational taxon (OTUs) with 97% similar truncation was clustered using UPARSE version 7.1, and the 16s rRNA database (Silva v138) was analyzed with RDP Classifier version 2.2. The confidence threshold is 0.7. The online platform of Majorbio i-Sanger Cloud platform (www.i-sanger.com) was used to analyze the 16S rRNA sequencing data.

Venn diagrams were used to count the number of species shared and unique in multiple groups of samples, which can show the similarity and overlap of species composition in the intestine of different groups of mice in a more visual way (Lu et al., 2016). Community barplot analysis was applied to quantify the abundance of species in the intestine of different groups of mice at different levels of taxonomy and the community composition to be studied visually. Hierarchical clustering of the distance matrix can clearly show the distance of different groups of sample branches, and according to different distance thresholds these samples can be divided into cohesive groups, thus mapping samples distances heatmap on Genus level. ANOSIM analysis, or Analysis of similarities (ANS) is a nonparametric test to test whether the differences in species composition in the intestine of different groups of mice are significantly greater than the within-group differences, and thus whether the grouping is meaningful. The Distance box plot was plotted with this result (Li et al., 2017). PLS-DA analysis, or Partial Least Squares Discriminant Analysis, is a multivariate statistical analysis method for discriminant analysis. It is a multivariate statistical analysis method used in discriminant analysis to determine how different samples are classified based on the observed or measured values of several variables (Wang et al., 2016). The test of significance of differences between groups was based on the community abundance data in the samples, and a strict Kruskal-Wallis rank sum was applied to detect species exhibiting differences in abundance in microbial communities in the gut of different groups of mice, and a hypothesis test was performed to assess the significance of the observed differences (Song et al., 2017). LEfSe firstly used the non-parametric factorial Kruskal-Wallis (KW) sum-rank test to detect features with significant abundance differences and to find taxa that differed significantly from abundance. Finally, linear discriminant analysis (LDA) was used to estimate the magnitude of the effect of abundance on the difference effect for each species (Guerrero-Preston et al., 2016).

### Statistical analysis

All experiments were repeated at least 3 times. All the data were analyzed and processed by GraphPad Prism 6.02 and SPSS 23.0 statistical software. Based on normality test and homogeneity of variance, Student-t test was used when two groups of data were compared (Welch’s t-test was used when the variance was not homogeneous). And one-way ANOVA analysis of variance was used when multiple groups of data were compared (Kruskal-Wallis test was used when the variance was not homogeneous).

## Results

### SH ameliorates histopathological changes in the lungs of mice induced by *P. aeruginosa*

**Figure 1:**
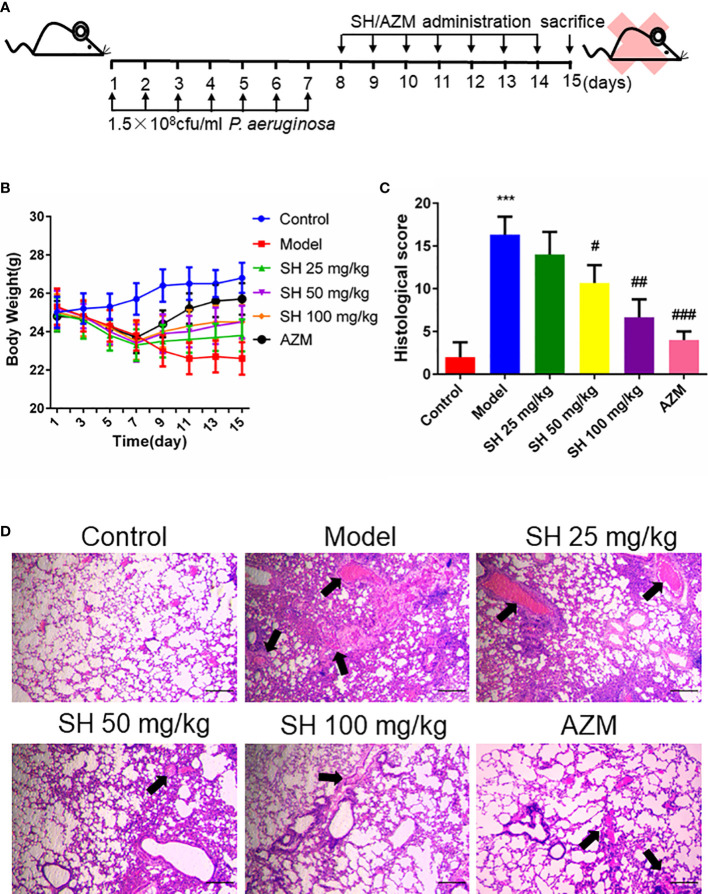
SH ameliorates histopathological changes in the lungs of mice induced by *P. aeruginosa*. **(A)** Experimental design of *P. aeruginosa* administration in pneumonia mice and SH treatment. **(B)** comparison of body weight of different treatment groups. **(C)** Histological scores. **(D)** Observe the pathological changes in the lung tissues of the mice by HE staining (×40 magnification). Scale bar, 20 µm. Arrows indicate reduced alveolar air space and infiltration of inflammatory cells. Data are represented as mean ± SD (n=3). ^***^P<0.001 mean significance of model group compared with control group. ^#^P<0.05, ^##^P<0.01 and ^###^P<0.001 mean significance of drug treatments compared with the model group.

Based on mouse model established by Allewelt and colleagues (Allewelt et al., 2000), we applied the pneumonia model in mice to investigate whether SH has an ameliorating effect on pneumonia in mice infected with *P. aeruginosa* (**Figure 1A**). Compared with the control group, the mice in the model group showed perinasal bleeding, weight loss caused by loss of appetite (**Figure 1B**), amount of inflammatory cell infiltration in lung tissue, destruction of alveolar wall structure, increased histological score (**Figure 1C**), and exfoliation of epithelial cells, diffuse red blood cells (**Figure 1D**) in the alveolar wall and alveolar cavity. After drug treatment, the appetite of mice in each drug-treated group increased and their body weight gradually recovered, while the model group did not improve. The weight gain of mice in AZM group was higher than that in SH groups, and there was no significant difference among SH groups (**Figure 1B**). After each drug treatment, the lung tissue structure of mice was relatively clear, and the infiltration of inflammatory cells around bronchioles and blood vessels decreased. There was no prominent lumen secretion in AZM group and SH 100 mg/kg group, and the histological score dropped significantly. A one-way ANOVA comparison result showed that there was a significant difference between the SH-treated group and the model group, that is, SH treatment could significantly improve lung inflammation in mice (**Figure 1C**). The above results suggest that SH can reduce the inflammatory damage in the lungs of mice caused by *P. aeruginosa*, namely, improve the pathological changes in the lungs of mice with pneumonia.

### SH reduces *P. aeruginosa* burden and systemic inflammation in pneumonia mice

**Figure 2:**
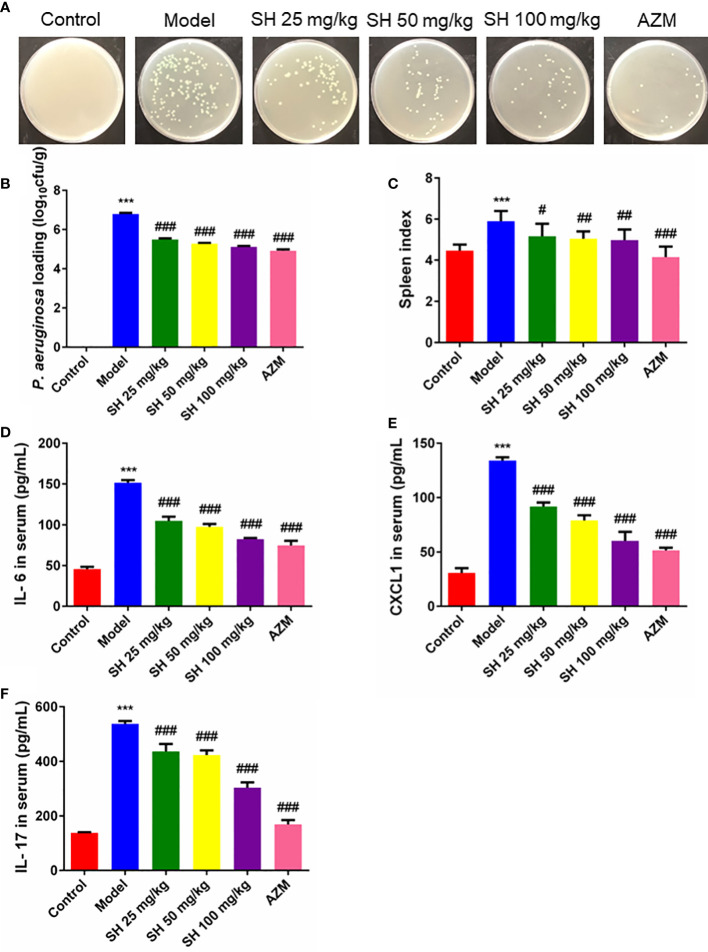
SH reduces *P. aeruginosa* burden and systemic inflammation in pneumonia mice. **(A)** The homogenized culture of mice lung tissue at different treatment after *P. aeruginosa* infection. **(B)** The number of viable *P. aeruginosa* isolated from the lung of mice. **(C)** Spleen index. **(D)** Serum IL-6, **(E)** CXCL1 and **(F)** IL-17 amounts were assessed by ELISA. Data are represented as mean ± SD (n=9). ^***^P<0.001 mean significance of model group compared with control group. ^#^P<0.05, ^##^P<0.01 and ^###^P<0.001 mean significance of drug treatments compared with the model group.

After seven days of infection, the amount of *P. aeruginosa* in the lung tissue homogenate was higher than 10^6^ colony forming units CFU/g in all groups except the control group. After seven days of drug treatment, the amount of *P. aeruginosa* in the lung tissue homogenate was higher than 10^5^ CFU/g in all treatment groups, except for the control group, where no *P. aeruginosa* colonies grew (**Figure 2A**). The results showed that the number of bacterial colonies in the SH treatment groups were significantly less than that in the model group in a dose-dependent manner (**Figure 2B**).

The spleen is an important immune organ in humans and animals (Zhang et al., 2019). Changes in spleen weight may be due to increased splenic function, congestion or infiltration and are therefore closely related to the immune response (Su et al., 2007; Ondee et al., 2017). Mice in the model group showed a significant increase in spleen index, suggesting that *P. aeruginosa* caused an inflammatory response *in vivo*, resulting in a significant increase in spleen mass. Compared with the model group, the spleen index of mice treated with each concentration of SH decreased significantly but did not reach the normal level. In contrast, that of the AZM group decreased significantly and reached the normal level (**Figure 2C**). Consistent with the above results, the pro-inflammatory cytokines IL-6, CXCL1 and IL-17 were significantly higher in the model group than in the control group. The levels of IL-6, CXCL1 and IL-17 at all concentrations were substantially lower in the SH and AZM groups than in the model group (**Figures 2D–F**). In addition, the pro-inflammatory cytokines IFN-γ and IL-25 were significantly higher in the model group than in the control group. The levels of IFN-γ and IL-25 were significantly lower in both the SH and AZM groups than in the model group, but were not dose-dependent, with the most significant effect of SH 50 mg/kg (**Figures S1A, B**).

Thus, these above results suggest that SH reduces the load of *P. aeruginosa* in the lungs of pneumonia mice, thus effectively interfering with acute pulmonary infection in mice. Meanwhile, SH can also reduce the inflammatory response of mice by inhibiting pro-inflammatory factors.

### SH ameliorates the expression of inflammatory genes and the production of inflammatory proteins in *P. aeruginosa* pneumonia mice

Pneumonia is characterized by the production of inflammation-related cytokines and chemokines throughout the body, especially in the lungs, including TNF- α, IFN- γ, IL-6 and IL-1β which are controlled by TLR4/NF-κB signaling pathway (Gaspar et al., 2013). Since detecting inflammatory factors directly in tissue samples is challenging, we used qRT-PCR to determine which relevant inflammatory factors were changed after SH treatment. And to clarify the function of SH on the TLR4/NF−κB signaling pathway, we detected the expression of p65, the phosphorylation of p65 (p-p65), inhibitor of NF-κB Alpha (IκB-α) and the phosphorylation of IκB-α (p-IκB-α) protein in tissues by western blotting.

**Figure 3:**
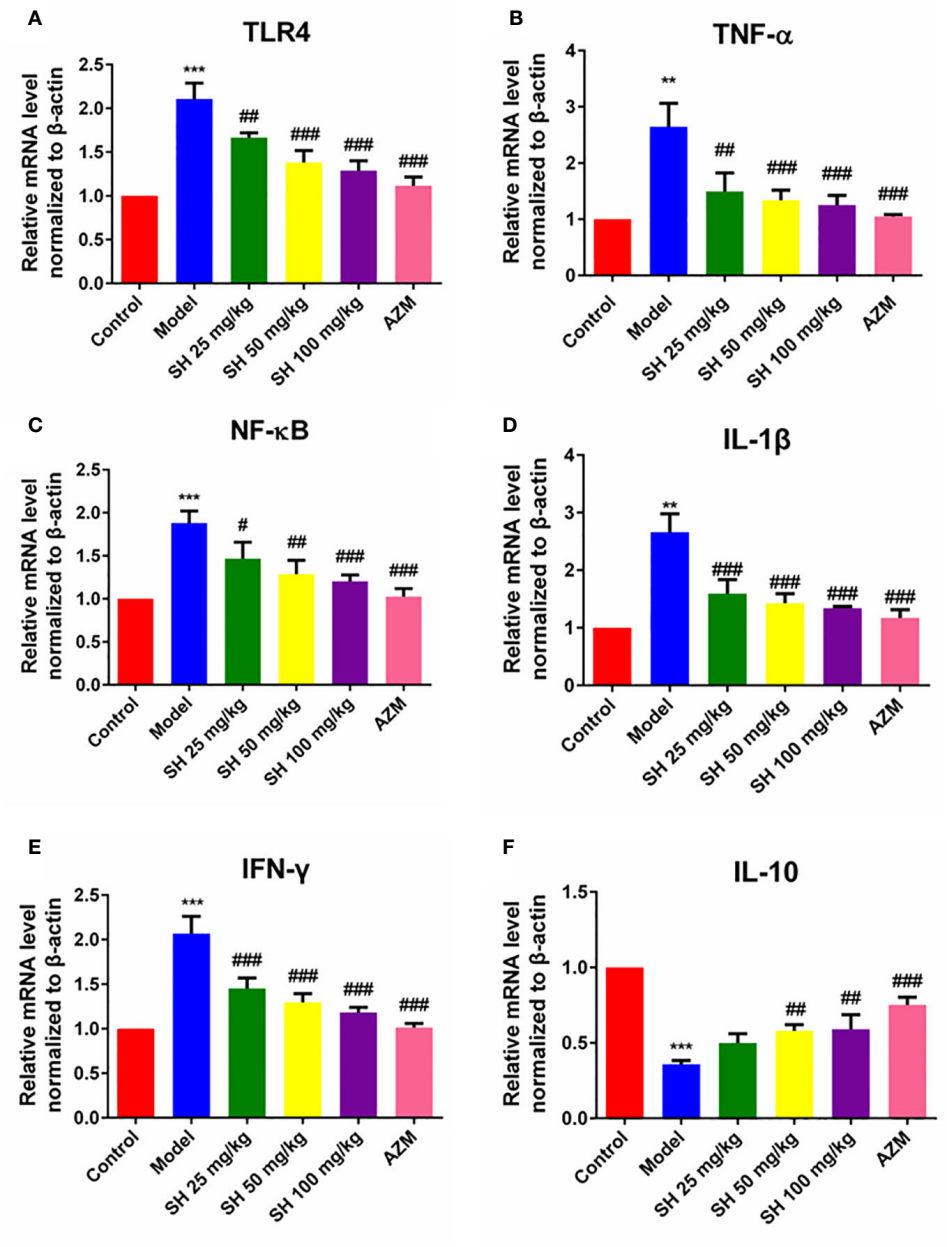
The expression of inflammatory genes in pneumonia mice after SH treatment. **(A)** *TLR4*. **(B)** *TNF-α*. **(C)** *NF-κB*. **(D)** *IL-1β*. **(E)** *IFN-γ*. **(F)** *IL-10*. Data are represented as mean ± SD (n=6). ^**^P<0.01 and ^***^P<0.001 mean significance of model group compared with control group. ^#^P<0.05, ^##^P<0.01 and ^###^P<0.001 mean significance of drug treatments compared with the model group.

**Figure 4:**
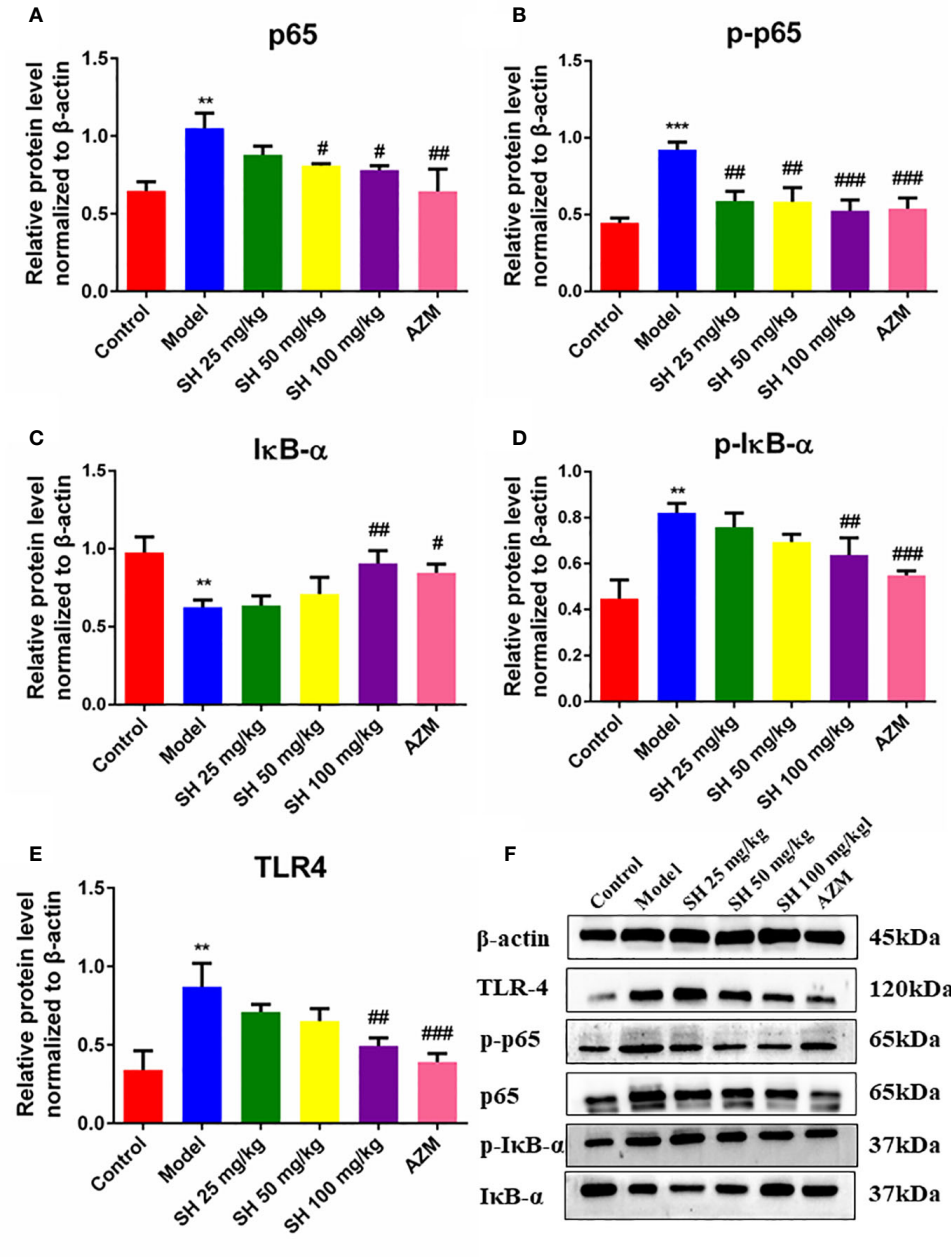
The expression of inflammatory proteins in pneumonia mice after SH treatment. **(A)** p65. **(B)** p-p65. **(C)** IκB-α. **(D)** p-IκB-α. **(E)** TLR4. **(F)** The expression of proteins. Staining intensity was assessed with Image J. Data are represented as mean ± SD (n=3). ^**^P<0.01 and ^***^P<0.001 mean significance of model group compared with control group. ^#^P<0.05, ^##^P<0.01 and ^###^P<0.001 mean significance of drug treatments compared with the model group.

QRT-PCR analysis (**Figures 3A–F**) showed that the expressions of *TLR4*, *NF-κB*, *TNF-α*, *IL-1β* and *IFN-γ* in the model group were significantly higher than those in the control group. In comparison, the expressions of *TLR4*, *NF-κB*, *TNF-α*, *IL-1β* and *IFN-γ* in the SH treatment groups were significantly lower than those in the model group. SH 100 mg/kg group had the best therapeutic effect but did not totally restore it to normal level. Compared with the control group, the expression of anti-inflammatory factor *IL-10* gene in the model group was significantly decreased. The expression of anti-inflammatory factor genes in each treatment group was dose-dependent and significantly higher than that in the model group. The results of western blotting (**Figures 4A–F**) revealed that the expressions of p65 (**Figure 4A**), p-p65 (**Figure 4B**), p-IκB-α (**Figure 4D**) and TLR4 (**Figure 4E**), in the model group were significantly higher than those in the control group. In the case of the SH treatment group, the relevant expression was significantly lower than that of the model group, especially in the SH 100 mg/kg group. The expression of IκB-α (**Figure 4C**) in the model group, was significantly lower than that in the control group, while the expression of IκB-α in the SH 100 mg/kg group was significantly higher than that in the control group. In addition, the expression of TNF-α (**Figure S1C**), IFN-γ (**Figure S1D**) and IL-1β (**Figure S1F**) in the model group was significantly higher than that in the control group. The expressions in the SH treatment group were significantly lower than those in the model group, especially in the SH 100 mg/kg group.

Taken together, these results suggest that SH treatment ameliorates *P. aeruginosa*-induced lung infection in mice by inhibiting the TLR4/NF-κB signaling pathway and suppressing pathway-related release of associated inflammatory factors.

### SH improves species composition of gut microbiota in pneumonia mice

Recently, increasing number of studies have revealed that intestinal flora is an effective target of drugs and regulates immunity to play the pharmacological role of anti-infection (Shao et al., 2020; Zhou et al., 2020). Therefore, we used high-throughput 16S rRNA sequencing technology to analyze the microbial diversity in the feces of each group of mice treated by SH.

**Figure 5:**
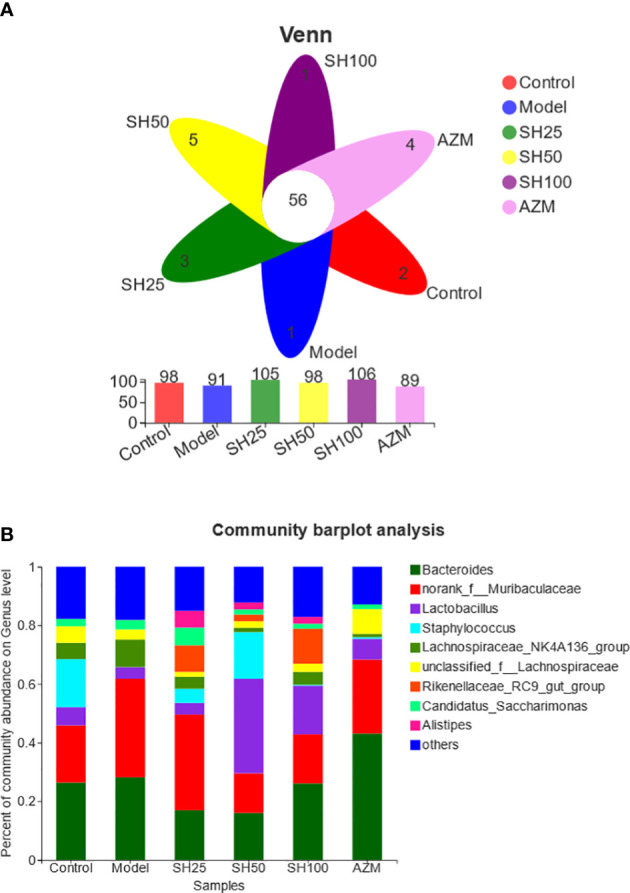
Analysis of species composition of gut microbiota. **(A)** Venn diagram: Different colors represent different populations (or samples), overlapping portions represent species common to multiple populations (or samples), non-overlapping portions represent species-specific to that population (or sample), and numbers indicate the corresponding number of species. **(B)** Community barplot histogram at genus level. Control group represents the control group without any treatment, Model, *P. aeruginosa* without any drug treatment. SH25, SH50 and SH100 mean SH 25 mg/kg, SH 50 mg/kg and SH 100 mg/kg.

Firstly, we analyzed the gut microbial species composition of the samples. In the Venn map (**Figure 5A**), 56 genera in 6 groups of samples overlapped with each other at Genus level, while 98 species, 91 species, 105 species, 98 species, 106 species and 89 species were differentially expressed in control, model, SH 25 mg/kg, SH 50 mg/kg, SH 100 mg/kg and AZM groups, respectively.

At the genus level, community barplot (**Figure 5B**) of the sample species relationship shows that the first 9 dominant bacteria in the 6 sample communities are *Bacteroides*, *Lactobacillus*, *Staphylococcus*, *Lachnospiraceae_NK4A136_group*, *unclassified_f_Lachnospiraceae*, *Rikenellaceae_RC9_gut_group*, *Candidatus_Saccharimonas*, *Alistipes* respectively. The proportion of dominant bacteria *Bacteroidtes* in control group was the highest, but the ratio of *Norank_f_Bacoidales* in model group was the highest. After drug treatment, SH 50 mg/kg and SH 100 mg/kg groups recovered the proportion of dominant bacteria. In addition, the three SH treatment groups differentially increased the number of *Lactobacillus*, *Rikenellaceae_RC9_gut_group* and *Alistipes* and decreased the number of *Bacteroides* compared to the model group.

Thus, the results shows that the dominant flora in the intestine of mice in the model group is altered and the number of beneficial bacteria is reduced compared to the control group, and the SH intervention can improve this phenomenon.

### SH improves alpha and beta diversity index in pneumonia mice

**Figure 6:**
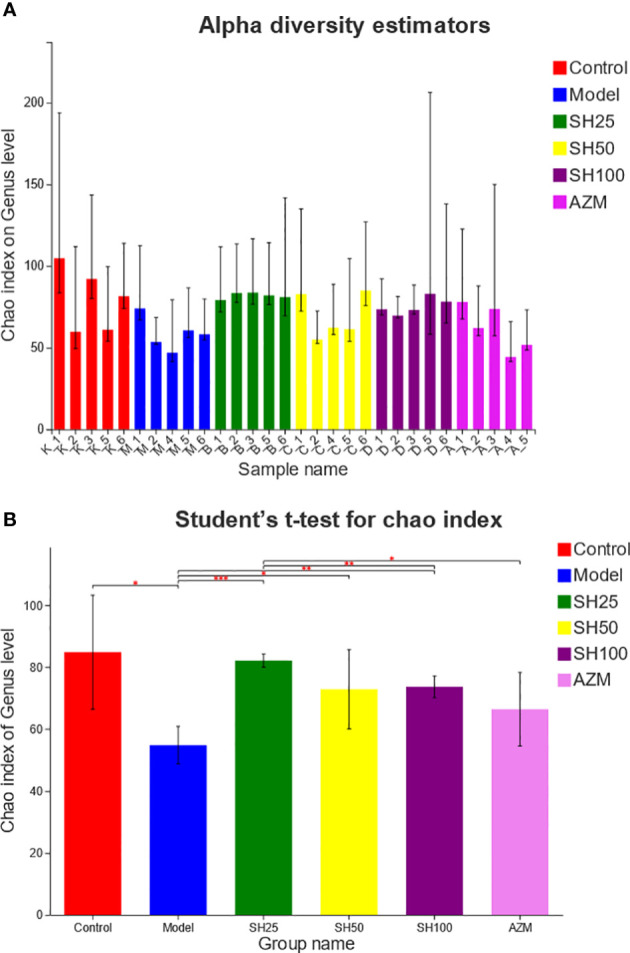
Alpha diversity analysis of gut microbiota. **(A)** Chao diversity index histogram. **(B)** Chao diversity test group t-test histogram. The X axe represents the group name, and The Y axe represents the exponential average of each group. ^*^0.001<P ≤ 0.05, ^**^0.001<P ≤ 0.01, ^***^P ≤ 0.001. SH25, SH50 and SH100 mean SH 25 mg/kg, SH 50 mg/kg and SH 100 mg/kg.

**Figure 7:**
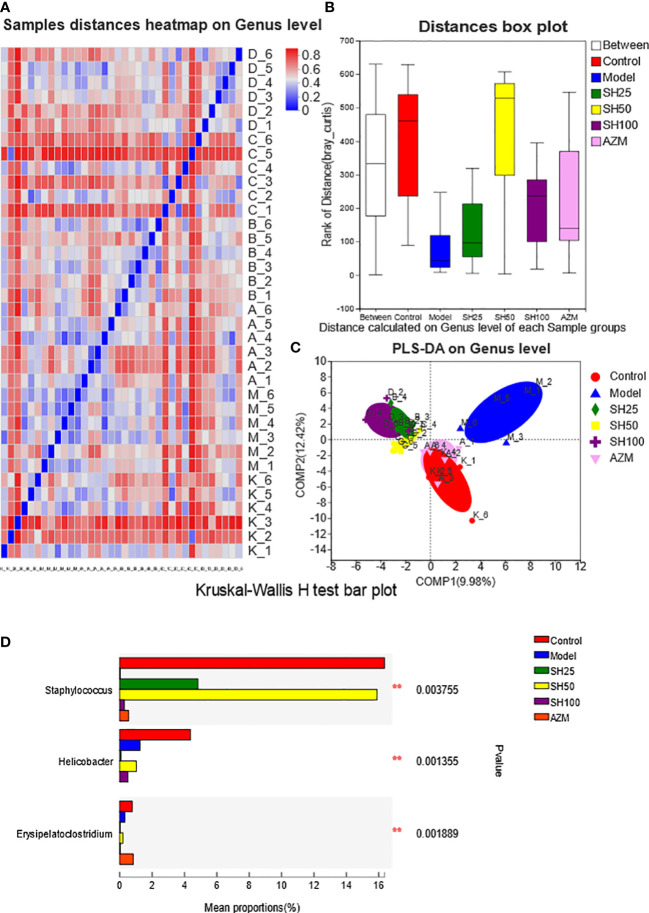
Analysis of comparison of gut microflora samples. **(A)** Sample distances heatmap on Genus level: The X and Y axes are samples, and the distance between samples is represented by different color gradients (the right side of the figure is the value represented by the color gradient). **(B)** Bray_Curtis analysis of similarities: The X axe represents the distance value within or Between groups, The Y axe represents the distance value, the box corresponding to Between represents the distance value Between groups, and the other boxes represent the distance value within the group, to analyze the explanation degree of different grouping factors for sample differences. **(C)** Partial least-squares discriminant analysis: Dots of different colors or shapes represent sample populations in different environments or conditions. The scale of the X and Y axes is relative distance and has no practical significance. Comp1 and Comp2 represent the suspected influencing factors of microbial composition bias of the two groups of samples, respectively. Kruskal-Wallis H-test bar plot analysis of differences in the abundance of intestinal flora species. **(D)** The vertical axis represents the species names at a taxonomic level. The column lengths corresponding to species indicate the average relative abundance of species in each sample group, and the different colors indicate the different groupings. ^**^0.001<P ≤ 0.01. SH25, SH50 and SH100 mean SH 25 mg/kg, SH 50 mg/kg and SH 100 mg/kg.

We compared and analyzed the alpha diversity index of the microbiota samples (**Figure 6**). The results showed that at genus level, the OTU richness (Chao index) of samples in the model group was significantly lower than that in the control group, and the OTU richness of samples in the SH treatment group was significantly restored, indicating that the intestinal microbial diversity of mice after SH treatment was higher than that of the model group. The heatmap diagram (**Figure 7A**) of sample hierarchical cluster analysis showed significant differences in the richness and diversity of microflora between the control group and the model group at the genus level, and these differences were reduced by SH treatment.

As shown by ANOSIM (**Figure 7B**), the Between value was more significant than the distance value of the other groups, and Bray_Curtis ANOSIM= 0.308318 score 0.001, indicating that the difference between the sample groups was more significant than that within the group, and the grouping was meaningful. In addition, the results showed (**Figure 7C**) that at the level of component 1 (9.98%) and component 2 (12.42%), the samples of the control group were closely arranged with those of the AZM group, and there were several overlaps. Compared with the AZM group, the distribution of the samples in the SH 25, SH 50 and SH 100 mg/kg groups was slightly farther from the control group but arranged closely with each other, and was significantly different from the model group. However, the PCA results showed that the sample locations of different groups were not distinct (**Figure S2A**). The results of flora typing analysis (**Figure S2B**) showed that the dominant flora of the control group was diversified, while the dominant flora of the model group was dominated by g_norank_f_Muribaculaceae. However, it is noteworthy that although the drug-treated group improved the dominant flora homogeneity of the model group, it did not return to the level of the control group.

According to the difference analysis of Chao index between groups, SH can improve the reduction of intestinal microbiota diversity in mice with pneumonia. And the results of the β-diversity PLS-DA analysis suggest that the overall composition of the intestinal flora is different in each group of mice, with the AZM group being the closest to the control group and the SH-treated group the next closest.

### SH reduces species abundance differences in pneumonia mice

Next, we conducted a species difference analysis to determine which species of bacteria in the intestinal tract had changed after drug treatment. Kruskal-Wallis H test bar plot (**Figure 7D**) showed that *Staphylococcus aureus* and *Erysipelato clostridium* in the model group were significantly lower than those in the control group. The former almost returned to the normal level after SH 50 mg/kg treatment, while the latter’s abundance was comparable to that of the normal group after AZM treatment. Moreover, *Helicobacter* in the model group was significantly lower than in the control group.

**Figure 8:**
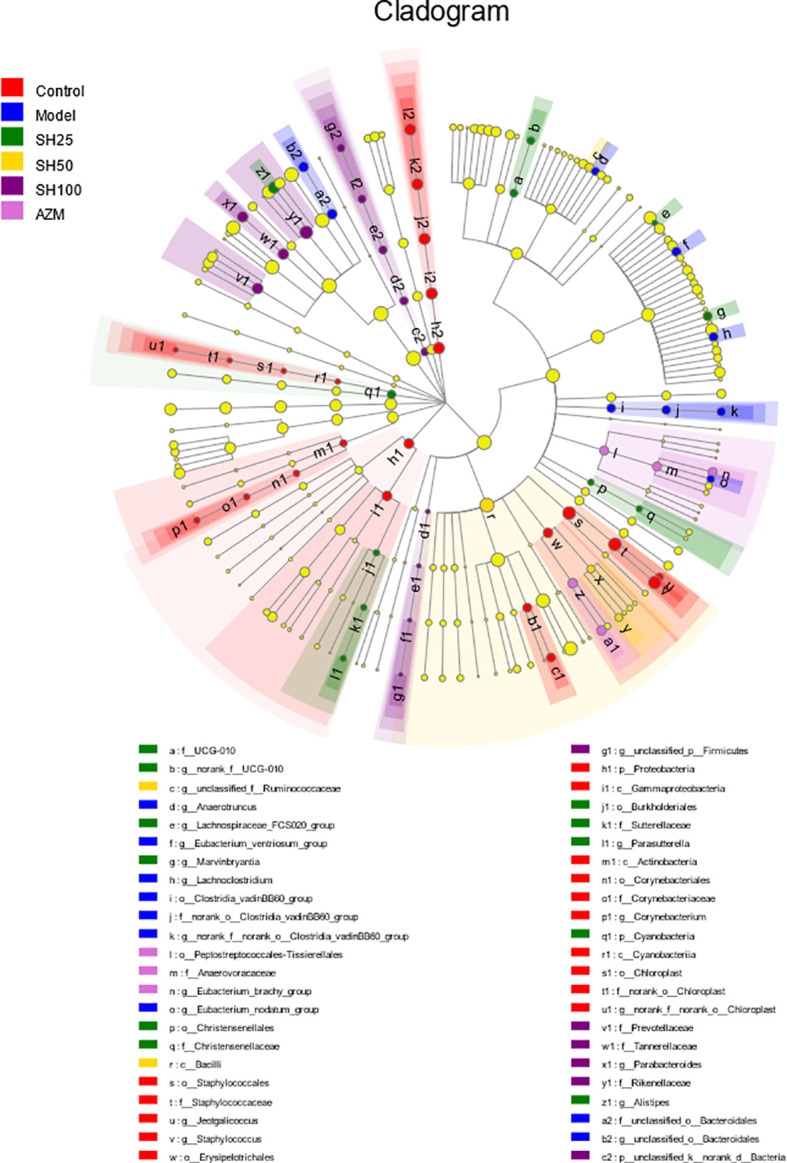
Multilevel species hierarchy tree diagram for LEfSe analysis. Different color nodes represent the microbial groups that are significantly enriched in the corresponding groups and significantly influence the differences between groups. The pale yellow nodes indicate the microbial groups that have no significant difference among different groups or have no significant effect on the difference between groups. SH25, SH50 and SH100 mean SH 25 mg/kg, SH 50 mg/kg and SH 100 mg/kg.

Similar to the results shown by Kruskal-Wallis H bars, LefSe analysis (**Figure 8**) showed that the control group had the highest number of nodes for abundance differences and the SH 100 mg/kg treatment group had the closest number of nodes to the control group. In contrast, the model group had the lowest number of nodes. This suggests that pneumonia can significantly reduce the abundance of intestinal microbiota in mice and that SH treatment can effectively increase the abundance of the flora to levels close to those of the control group, which is similar to the AZM group.

**Figure 9:**
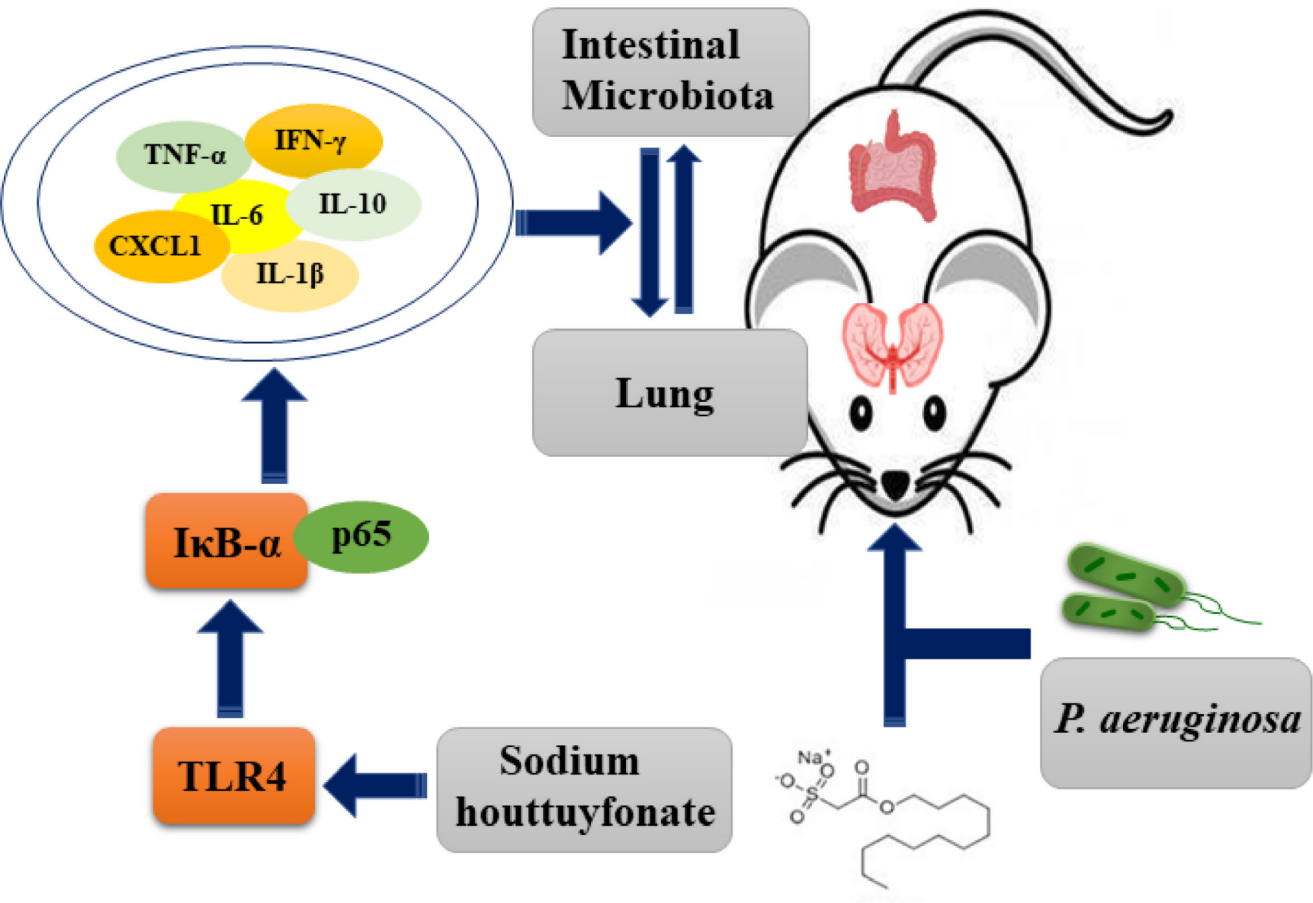
Schematic diagram of the possible mechanism of sodium hottuyfonate against acute pulmonary infection by *P. aeruginosa*. SH can regulate the immune and intestinal flora disorders of mice through TLR4/NF-κB signaling pathway, and effectively alleviate acute lung infection caused by *P. aeruginosa*.

Combining the above results, we can conclude that *P. aeruginosa*-induced pneumonia in mice can reduce the diversity of intestinal flora and substantially reduce its abundance (**Figure 9**). In addition, the presented results indicate that the ratio and abundance of various bacteria in the flora can be significantly restored by SH treatment.

## Discussion

*Houttuynia cordata* is a traditional Chinese herb that has been shown to possess potent activity to treat infectious diseases (Gao et al., 2010; Shingnaisui et al., 2018; Wu et al., 2021). SH is the most crucial active component of *Houttuynia cordata*, widely used in anti-infection. In previous studies, we found that although SH was effective in inhibiting the growth of *P. aeruginosa* biofilm and eliminating its adhesion, SH could only inhibit the growth of bacteria and could not kill them directly (Wu et al., 2014). Thus, SH *in vivo* may achieve anti-infective effects by enhancing the immune response and altering gut microbes, rather than directly antimicrobial killing microbes as antibiotics do. Since the mechanism of action of SH against infection *in vivo* is not clear, we evaluated the protective effect of SH *in vivo* in a mouse acute pneumonia model.

After homogenization of lung tissues, the number of bacteria in the model group was higher than 10^6^ CFU/g, thus successfully establishing a mouse pneumonia model induced by *P. aeruginosa* nasal drip. The results of this study show that SH improves the general status of mice with increased body weight and also improves the histological score of lung tissue in mice with *P. aeruginosa*-induced pneumonia. The spleen index is a preliminary observation characterizing the immune function of the organism and can be used to evaluate the effect of drugs on the immune organs (Li and Guan, 2011). Therefore, the results of the measurement of the splenic index of the immune organs of mice indicate that SH treatment can effectively control the deterioration of *P. aeruginosa* infection.

Inflammatory factors play an essential role in the occurrence and development of pneumonia. When the organism is attacked by *P. aeruginosa*, the expression of multiple inflammatory factors is increased or decreased to varying degrees (Zuercher et al., 2006; Alipour et al., 2013; Chen et al., 2018). Lipopolysaccharide (LPS), a major component of the outer membrane of *P. aeruginosa*, stimulates TLR4, and the TLR4 pathway activates NF-κB (Liu et al., 2015). Usually, in unstimulated cells, NF-κB is located in the cytoplasm and binds to its IκB-α receptor (Zhu et al., 2016). Once the cells are treated with an inducer, inhibitor of NF-κB (IκB) is degraded and phosphorylated NF-κB p65 is transferred to the nucleus (Lawrence, 2009). Finally, it triggers the release of various pro-inflammatory cytokines, such as TNF-α, IL-1β, IL-6 and CXCL1 (Lawrence et al., 2005). In the study by Wang et al. (Wang et al., 2017), LPS caused phosphorylation of NF-κB p65 and phosphorylation of IκB, whereas SH treatment significantly inhibited LPS-induced activation of NF-κB, thereby regulating the expression of related inflammatory cytokines. In this study, our results showed that SH significantly inhibited TLR4 expression in the lungs of *P. aeruginosa*-infected mice while causing phosphorylation of NF-κB p65 and phosphorylation of IκB-α, while SH suppressed the related expression thereby inhibiting the TLR4/NF-κB signaling pathway. Under normal conditions, a dynamic balance of pro-inflammatory and anti-inflammatory cytokines is maintained, and infection can lead to an alteration of this balance, which may be crucial for the pathogenesis of inflammatory diseases. Zhang and colleagues (Zhang et al., 2020) found that SH reduced the inflammatory response induced by *Salmonella typhimurium* through the NF-κB pathway. Expression of IL-6 and CXCL1 in serum and NF-κB, TLR4 and IL-1β in lung tissue were significantly higher in their model group than in the control group and decreased significantly in a dose-dependent manner after SH treatment. Similar results were found in our study, where multiple inflammatory cytokines in *P. aeruginosa*-induced pneumonia mice produced significant differences after SH treatment, that is, SH intervention reduced the release of each of these pro-inflammatory cytokines. Moderate inflammation is beneficial and protective, while both excessive and insufficient inflammation is detrimental to the body (Chen et al., 2022). Compared to the model group, SH reduces the inflammatory response in the body and maintains it at an appropriate level, which may ensure that the body is functioning properly in draining pathogenic bacteria and toxins, controlling the inflammatory response, and avoiding the waterfall effect and severe self-injury. Notably, our previous study indicate that long term and high dose of SH (usage of three week and 400 mg/kg) can increase the production of NF-κB and IFN-γ, indicating the overuse of SH can affects the composition of gut microbiota and production of inflammatory factors to induce pathological damage potentially in mice (Yan et al., 2020). Thus, based on the previous research, we set the concentration of SH of the high dosage, moderate dosage, low dosage as 100 mg/Kg, 50 mg/Kg and 25 mg/Kg and drug use of time as 1 week in the present research to avoid the potential pathological damage effect. In addition, instead of KM mice which have a complex genetic background, we select specific pathogen-free (SPF) BALB/c mice which have a clear genetic background and widely used in the pharmacological research (Wang et al., 2016; Yu et al., 2016). Combine these, our present research may obtain reliable results on the effect and possible mechanism of SH against the pneumonia induced by *P. aeruginosa* in mice.

In recent years, intestinal flora has been recognized as an important indicator of the pharmacological effects of traditional Chinese medicine (Feng et al., 2019). The intestinal flora can modulate the immune response of the mucosal immune system under normal physiological and infectious states (Hills et al., 2019). Due to the ability of the flora and its metabolites to mediate mucosal immunity, when the abundance and number of bacteria fluctuate, they inevitably affect the secretion of cytokines and immunoglobulins, thus exacerbating the inflammatory process (Fagundes et al., 2012). However, the mechanisms by which intestinal flora are associated with pulmonary immunity have not been elucidated, and current studies suggest that they may be related to the activation of Toll-like receptors (TLRs) (Lowe et al., 2010; Jiminez et al., 2016). Tang and colleagues (Tang et al., 2021) found that dysbiosis of the gut microflora could exacerbate endotoxin-induced acute lung injury by activating the TLR4/NF-κB signaling pathway in the lung and antagonize LPS-induced acute lung injury by restoring the intestinal flora and improving the diversity of the intestinal flora through fecal transplantation. The results of this study are consistent to our study, in which we found that SH significantly ameliorated *P. aeruginosa* lung inflammation through activation of the TLR4/NF-κB signaling pathway and improved intestinal flora dysbiosis. Here, we found that SH intervention restored the dominant species and increased the proportion of beneficial flora, and the three SH treatment groups increased the number of *Lactobacillus*, *Rikenellaceae_RC9_gut_group* and *Alistipes* to different degrees. *Lactobacillus* can improve ventilator-associated pneumonia (Johnstone et al., 2021), and *Rikenellaceae_RC9_gut_group* are positively correlated with Butyric and valeric acids (Qing et al., 2019), and *Alistipes* may have a protective effect against certain diseases (Parker et al., 2020). Although the exact mechanism of their rise in numbers is not clear, it is possible that SH increases intestinal flora by stimulating intestinal protective mechanisms, the mechanism of which needs further investigation. In addition, the results of β-diversity analysis showed that the overall composition of the intestinal flora in each group of mice was closest to that of the control group in the AZM group, followed by SH 50 mg/kg. The results of LefSe analysis also showed that the abundance of intestinal flora was significantly lower in the model group compared with the control group, while the abundance of various bacteria in the flora could be significantly restored after SH treatment. Thus, our results imply that SH could improve intestinal flora dysbiosis and potentially modulate the immune system in mice model of pneumonia induced by *P. aeruginosa*.

## Conclusion

Collectively, our presented results suggest that SH may effectively treat *P. aeruginosa*-induced pneumonia in mice through the TLR4/NF-κB signaling pathway modulating immunity and affecting intestinal flora. However, there are some limitations of the current study. Such as the detail mechanism of how the intestinal flora and its bacterial metabolites regulate the inflammatory response in the lung has not been investigated in depth. Therefore, more experiments are needed to validate the above questions in our further research.

## Data availability statement

The datasets presented in this study can be found in online repositories. The names of the repository/repositories and accession number(s) can be found below: https://www.ncbi.nlm.nih.gov/, PRJNA781056.

## Ethics statement

The animal study was reviewed and approved by Laboratory Animal Ethics Committee of Anhui University of Chinese Medicine.

## Author contributions

DW, JW, and LM contributed to conception and design. TZ and MH performed animal experiments. XZ, HZ and FJ were involved in data analysis. JS, TW and CW provided the resources. TZ and MH drafted the manuscript. DW and XN did a critical revision of the manuscript. DW supervised the article. All authors contributed to the article and approved the submitted version.

## Funding

This work was supported by the projects funded by Excellent Young Talents Fund Program of Higher Education Institutions of Anhui Province (grant numbers gxyqZD2020024); the Natural Science Foundation (Key project) of University in Anhui province (grant numbers KJ2020A0441); and the Natural Science Foundation (Key project) of Anhui University of Chinese Medicine (grant numbers 2020zrzd07).

## Acknowledgments

The authors thank Shanghai Meiji biomedical technology co., ltd. for the assistance of 16S rRNA sequencing and bioinformatics analysis.

## Conflict of interest

The authors declare that the research was conducted in the absence of any commercial or financial relationships that could be construed as a potential conflict of interest.

## Publisher’s note

All claims expressed in this article are solely those of the authors and do not necessarily represent those of their affiliated organizations, or those of the publisher, the editors and the reviewers. Any product that may be evaluated in this article, or claim that may be made by its manufacturer, is not guaranteed or endorsed by the publisher.

## Abbreviations

AZM, azithromycin; BCA, bicinchoninic acid; cDNA, complementary DNA; CFU, colony forming units; CXCL1, C-X-C motif chemokine ligand 1; H & E, hematoxylin-eosin; IFN-γ, Interferon-γ; LB, Luria-Bertani; IL-1β, interleukin-1β; IL-10, interleukin-10; IL-6, interleukin-6; IκBα, inhibitor of NF-κB Alpha; IκB, inhibitor of NF-κB; LPS, Lipopolysaccharide; NF-κB, Nuclear Factor kappa B; *P. aeruginosa*, *Pseudomonas aeruginosa*; PBS, phosphate buffered saline; PMSF, phenylmethanesulfonyl fluoride; PVDF, polyvinylidene difluoride; RIPA, radioimmunoprecipitation assay; SH, Sodium houttuyfonate; SNH, sodium new houttuyfonate; SPF, specific pathogen-free; TBS, tris buffered saline; TLR4, Toll−like receptor 4; TLRs, Toll-like receptors; TNF-α, tumor necrosis factor-α.
