# Supplementary figures and images for: Sodium houttuyfonate effectively treats acute pulmonary infection of Pseudomonas aeruginosa by affecting immunity and intestinal flora in mice
Source: Front Cell Infect Microbiol. 2022 Dec 1;12:1022511. doi: 10.3389/fcimb.2022.1022511 (PMC9751016; doi:10.3389/fcimb.2022.1022511)

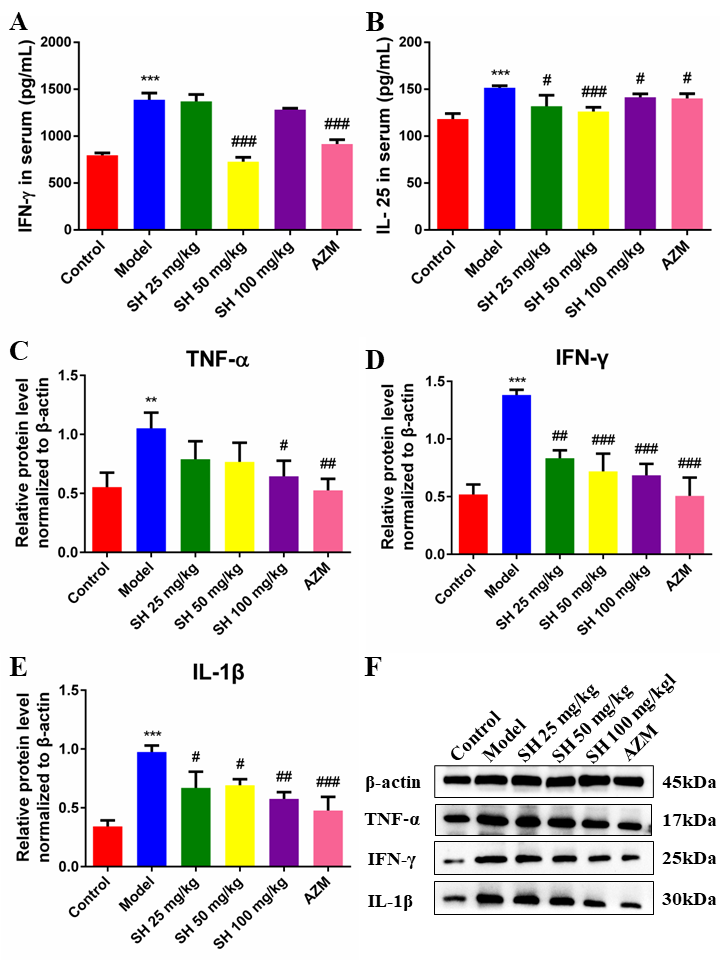

Supplement: Supplementary file 1 [file Image_1.tif]

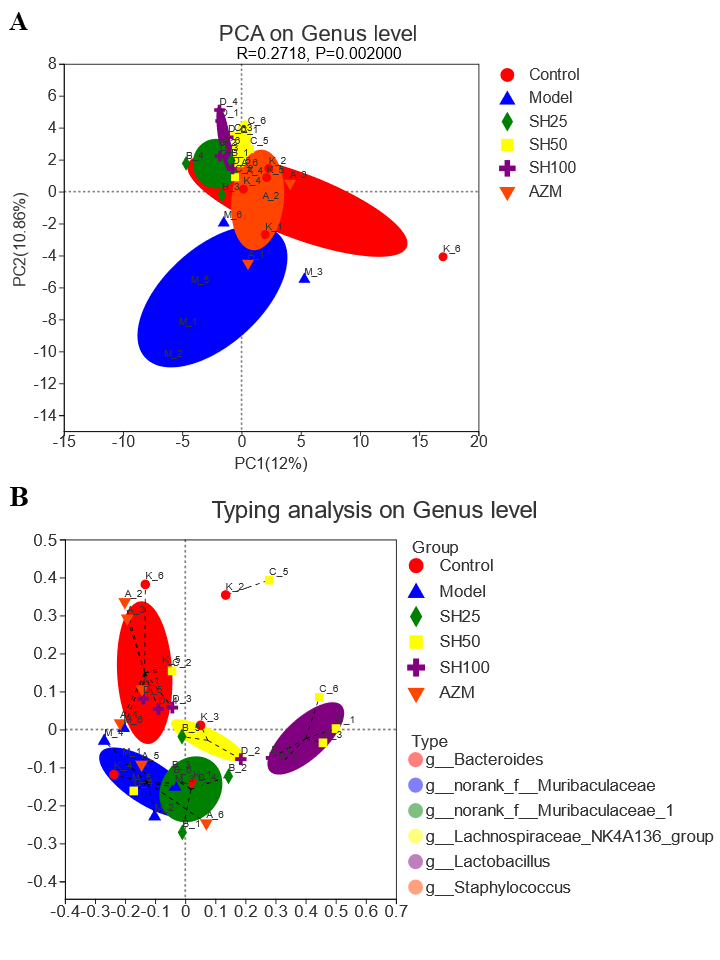

Supplement: Supplementary file 2 [file Image_2.tif]
